# Epidemiological Analysis of Rabies Outbreaks in the European Union and Türkiye (2013–2023)

**DOI:** 10.3390/life16060877

**Published:** 2026-05-24

**Authors:** Ralitsa Rankova, Dilek Muz, Koycho Koev, Gergana Balieva

**Affiliations:** 1 Department of Food Quality and Safety and Veterinary Legislation, Faculty of Veterinary Medicine, Trakia University, 6000 Stara Zagora, Bulgaria; ralitsa.rankova@trakia-uni.bg (R.R.); gergana.balieva@trakia-uni.bg (G.B.); 2 Department of Virology, Faculty of Veterinary Medicine, Tekirdag Namik Kemal University, 1504 Tekirdag, Türkiye; dilekmuz@nku.edu.tr; 3 Department of Veterinary Microbiology, Infectious and Parasitic Diseases, Faculty of Veterinary Medicine, Trakia University, 6000 Stara Zagora, Bulgaria

**Keywords:** rabies, zoonotic disease, epidemiology, animal disease surveillance, wildlife reservoirs, vaccination programs, Europe, Türkiye

## Abstract

Rabies is a fatal zoonotic viral disease that continues to pose a significant threat to both animal and public health worldwide. Despite considerable progress in its control across Europe, sporadic outbreaks still occur, particularly in regions where wildlife reservoirs and stray animal populations sustain virus circulation. This study provides one of the first comparative longitudinal analyses integrating European countries and Turkiye rabies surveillance data over a decade (2013–2023). Information on reported outbreaks was obtained from the Animal Disease Information System (ADIS) and the World Animal Health Information System (WAHIS) database. The analysis focused on temporal trends, regional differences, and the distribution of affected animal species. During the study period, a total of 4865 outbreaks were reported in 16 countries. The number of detected outbreaks declined considerably over time, decreasing from 1022 cases in 2013 to 325 cases in 2023, representing an overall reduction of approximately 68%. The temporal trend was not uniform, with periods of decline followed by temporary increases. The highest number of outbreaks was registered in Türkiye, followed by Romania and Poland, indicating pronounced regional disparities. Domestic dogs represented the most frequently affected species, while cases were also recorded in wildlife and domestic cats, confirming the epidemiological importance of both domestic and wild reservoirs. The observed reduction in the number of outbreaks reflects the impact of vaccination programs and coordinated control measures, but may also be influenced by differences in surveillance systems and reporting practices. Nevertheless, the persistence of rabies in several regions indicates that the disease remains an epidemiological concern. Sustained vaccination of domestic animals, continued wildlife immunization, and strengthened surveillance and cross-border cooperation are essential for long-term control and prevention.

## 1. Introduction

Rabies is a deadly, highly infectious zoonotic viral disease that poses a serious threat to both humans and animals worldwide. The causative agent, Rabies virus (RABV), is a negative-sense RNA virus classified within the *Lyssavirus* genus of the *Rhabdoviridae* family, under the order *Mononegavirales*. RABV is a neurotropic virus transmitted primarily through bites or wounds and spreads via peripheral nerves to the central nervous system, resulting in a rapidly progressing disease with an almost invariably fatal outcome once clinical signs appear [1]. Due to its zoonotic nature, rabies represents a substantial public health threat both in Europe and globally [2]. The disease is generally described in encephalitic (furious) and paralytic forms, progressing through stages that ultimately lead to death. The incubation period varies depending on factors such as bite location, wound severity, and viral load [3]. Although no effective treatment exists after the onset of symptoms, prompt administration of rabies vaccine and human rabies immune globulin (HRIG) can prevent a fatal outcome [4,5]. Rabies virus affects all mammals, including domestic animals such as dogs, cats, cattle, horses, and sheep, as well as wildlife reservoirs such as bats, raccoons, foxes, skunks, and mongooses [6]. Domestic dogs are the main transmitters of rabies to humans, accounting for approximately 90% of global human rabies deaths. Rabies remains a significant global health problem, causing an estimated 30,000 to 59,000 deaths annually [7], with approximately 95% of cases occurring in Asia and Africa [8,9].

Domestic cats represent a significant share of incidental hosts of RABV in their respective regions [10,11]. Although they are reported less frequently than dogs, they are included in mandatory vaccination programs in many countries [12]. It is believed that rabies in cats may be underreported in several regions. Nevertheless, rabid cats have been documented across all inhabited continents except Australia [13,14], and recent years have seen an increase in human exposure associated with infected cats [15,16]. Clinical signs of rabies in felines may overlap with those of other neurological conditions, which can complicate differential diagnosis. For example, infections such as Toxoplasma gondii may present with similar neurological manifestations; however, studies have demonstrated that animals positive for Toxoplasma gondii tested negative for rabies despite exhibiting comparable clinical signs [17]. These findings highlight the importance of accurate laboratory diagnosis in distinguishing rabies from other neurological diseases.

From an epidemiological perspective, ruminants may contribute to the spread of rabies and represent a potential source of occupational exposure for veterinarians, slaughterhouse workers, animal owners, and handlers involved in the management of infected animals [18,19,20]. In addition, the potential entry of infected animals into the food chain highlights the importance of raising awareness among relevant stakeholders. Improving public awareness is essential for effective disease control and food safety, particularly in situations where rabies-infected ruminants may be slaughtered [21,22].

Assessing risk factors related to the transmission and persistence of the rabies virus in both wildlife and domestic animal populations is essential for improving control measures. Studies analyzing the spatial and temporal distribution of rabies cases have demonstrated the key role of wildlife reservoirs, particularly red foxes, which account for a large proportion of reported cases [23]. This evidence highlights the importance of understanding wildlife population distribution and density for the development of effective targeted vaccination strategies. Additional epidemiological analyses have identified spatial clustering of rabies cases among species such as raccoons, foxes, and marmots, with geographical features such as rivers and lakes acting as natural barriers that may limit disease spread [24]. These findings emphasize the importance of targeted wildlife vaccination programs for effective rabies control, reducing transmission, and minimizing public health and economic impacts [25].

Timely and accurate diagnosis of rabies in both humans and animals is essential due to the nearly 100% mortality rate once clinical signs appear and its importance for effective disease management and prevention. Diagnostic methods for rabies vary in performance, and differences in sensitivity and specificity may affect case detection. Although techniques such as ELISA and RT-PCR are widely used, variability in diagnostic accuracy highlights the need for reliable and standardized approaches. Improvements in diagnostic technologies can reduce false-negative results and contribute to more effective rabies surveillance and control [26].

Rabies is also classified as a neglected tropical disease and remains a major global threat to both human and animal health, particularly in regions with limited resources. A One Health approach, based on coordinated efforts between the medical and veterinary sectors, is essential for effective rabies control and prevention. Such collaboration supports the global goal of eliminating rabies as a public health threat by 2030, as defined by the World Health Organization [27]. This objective is further reinforced by the 2018 Tripartite Memorandum of Understanding between WHO, FAO, and OIE, which promotes joint actions at the human–animal–environment interface [28].

Despite these efforts, important gaps remain in the understanding of regional epidemiological patterns, particularly in relation to differences between European Union countries and neighboring regions such as Türkiye. The aim of the present study was to analyze the temporal trends, spatial distribution, and species-specific patterns of rabies outbreaks in Europe and Türkiye over the period 2013–2023, and to provide a comparative assessment of regional epidemiological differences.

## 2. Materials and Methods

### 2.1. Study Design and Data Sources

This study analyzes rabies outbreaks reported in animals in European countries and Türkiye over the period 2013–2023, using officially available epidemiological data. The analysis was based on information obtained from international and European animal disease reporting systems. Data were obtained from the Animal Disease Information System (ADIS; European Commission, Brussels, Belgium; https://webgate.ec.europa.eu/tracesnt/adis/public/notification, accessed on 15 January 2026) and the WOAH World Animal Health Information System (WAHIS; World Organisation for Animal Health, Paris, France; https://wahis.woah.org/). These systems record official notifications submitted by national veterinary authorities and provide information on notifiable animal diseases, including the number of outbreaks, affected species, and the reporting country. Both systems are based on official national notifications and therefore share a common primary data source. Substantial overlap may exist between ADIS and WAHIS records, as the same outbreaks can be reported in both systems. However, differences in data aggregation, temporal resolution, and reporting practices may result in inconsistencies between the datasets. For the purposes of quantitative analysis, a single primary data source was used to ensure consistency and to avoid potential duplication of reported outbreaks. To avoid duplication, only one primary data source (ADIS) was used for quantitative analysis, and no merging of records between ADIS and WAHIS was performed. Cross-checking between ADIS and WAHIS was performed at an exploratory level to assess general consistency between the datasets; however, due to differences in reporting structure, only one dataset was used for formal analysis.

In addition to ADIS outbreak data, complementary official surveillance sources were consulted to address animal-species distribution in Türkiye, human rabies cases, and bat-associated lyssavirus data. Species-resolved animal rabies data for Türkiye were extracted from the WHO Rabies Bulletin Europe database (WHO Collaborating Centre for Rabies Surveillance and Research, Friedrich-Loeffler-Institut, Greifswald–Insel Riems, Germany; https://www.who-rabies-bulletin.org/); although the query covered 2013–2023, species-level records were available only for 2013–2019. Human rabies data were reviewed using WHO Global Health Observatory (World Health Organization, Geneva, Switzerland; https://www.who.int/data/gho), WHO Rabies Bulletin Europe, ECDC Annual Epidemiological Reports (European Centre for Disease Prevention and Control, Solna, Sweden; https://www.ecdc.europa.eu/en/publications-data/monitoring/all-annual-epidemiological-reports, accessed on 15 January 2026), and EFSA/ECDC One Health Zoonoses Reports (European Food Safety Authority, Parma, Italy; European Centre for Disease Prevention and Control, Solna, Sweden; https://www.efsa.europa.eu/; https://www.ecdc.europa.eu/) to provide context on public health context, without statistical comparison with animal outbreak data. Because ADIS did not provide bat-specific information, WHO Rabies Bulletin Europe, ECDC Annual Epidemiological Reports, EFSA/ECDC One Health Zoonoses Reports, WOAH/WAHIS public outputs (World Organisation for Animal Health, Paris, France; https://wahis.woah.org/), and relevant published literature were consulted to assess whether bat-associated lyssaviruses could be quantitatively addressed during the study period.

### 2.2. Inclusion Criteria and Data Collection

For the purposes of the study, all officially reported rabies outbreaks in animals within the selected geographical area and time period (2013–2023) were included. All reported outbreaks were included regardless of animal species, provided they were officially notified within the study period. No additional inclusion or exclusion criteria were applied beyond those defined by the reporting systems. The collected data were organized according to year, country, and animal species involved in the reported outbreaks. Data were systematically extracted at the level of country, year, and affected animal species from the selected database. Records with missing information were retained in the dataset but were not included in analyses requiring complete data. Geographical maps were obtained directly from the Animal Disease Information System (ADIS) and are presented as provided by the database. These visualizations reflect officially reported outbreak data and were not modified for the purposes of this study.

### 2.3. Definition of Outbreak

The definition of a rabies outbreak followed the criteria used by the reporting systems (ADIS and WAHIS), which are based on official national notifications. However, differences in outbreak definitions and reporting practices between countries may affect the comparability of the data. In general, an outbreak refers to one or more confirmed cases of rabies in animals within a defined epidemiological unit, as reported by national veterinary authorities.

### 2.4. Data Analysis and Epidemiological Approach

The analysis focused on evaluating temporal changes in the number of reported rabies outbreaks and identifying the animal species most commonly affected. Particular attention was given to the role of domestic animals and wildlife in the epidemiology of the disease. Descriptive summaries were used to illustrate the temporal and spatial distribution of rabies outbreaks across the study region. Comparative analyses between Türkiye and European Union countries were performed using descriptive statistical approaches, based on annual outbreak counts and the relative contribution of each region to the total number of reported outbreaks. Stratified analyses were conducted by grouping the data according to animal category (domestic versus wildlife) and country-level burden, allowing qualitative comparison of epidemiological patterns across different groups. Species-level analysis for Türkiye was performed using WHO Rabies Bulletin Europe data for 2013–2019, based on the available official species-resolved records. These approaches were applied to enhance the analytical depth of the study beyond purely descriptive statistics. Potential limitations of the dataset include underreporting, differences in surveillance capacity between countries, and variations in diagnostic capabilities over time, which were considered in the interpretation of the results.

### 2.5. Statistical Analysis

Statistical analysis of the collected data was performed using IBM SPSS Statistics (version 26, IBM Corporation, Armonk, NY, USA). The dataset included the number of rabies outbreaks reported annually for each country during the study period (2013–2023). Descriptive statistical methods were used to summarize the distribution of outbreaks across years and countries. Continuous variables were expressed as mean, median, standard deviation (SD), minimum, and maximum values, while categorical variables were presented as frequencies and percentages. The relative contribution of each country to the total number of reported outbreaks was calculated as a percentage of the overall dataset. To evaluate the temporal dynamics of rabies outbreaks, linear regression analysis was performed, with year as the independent variable and the total number of outbreaks as the dependent variable. The strength and direction of the association between time and outbreak occurrence were assessed using the Pearson correlation coefficient (r) and the coefficient of determination (R^2^). All statistical tests were two-tailed, and *p*-values < 0.05 were considered statistically significant.

The assumptions of linear regression, including linearity, independence of observations, and normality of residuals, were evaluated prior to analysis. Residuals were further examined to assess approximate normality and homoscedasticity. Given the aggregated nature of the data and the observed year-to-year fluctuations, the limitations of applying a linear model to epidemiological data were considered, and the results of the regression analysis were interpreted with caution.

## 3. Results

### 3.1. Overall Distribution

Rabies is a notifiable disease under the European Union Animal Health Law and related implementing regulations [29,30,31], which define the framework for disease surveillance, reporting, and control within Member States. The distribution of reported rabies outbreaks by country and year over the study period (2013–2023) is presented in Table 1.

A total of 4865 rabies outbreaks were reported during the study period. The data demonstrate a strong geographical concentration of outbreaks, with Türkiye accounting for 68.26% of all reported cases, followed by Romania (14.22%) and Poland (11.61%). This pattern indicates that the epidemiological burden of rabies is highly uneven across the study region, with a limited number of countries contributing the majority of reported outbreaks. A clear temporal decline in outbreak numbers was observed over the study period. The number of reported outbreaks decreased from 1022 in 2013 to 325 in 2023, representing an approximate 68% reduction. However, the temporal trend was not linear. A pronounced decline occurred between 2013 and 2016, followed by a period of fluctuation and relative stabilization from 2017 onwards. These fluctuations suggest that rabies dynamics varied over time rather than following a consistent decreasing pattern.

Based on the cumulative number of outbreaks, Türkiye, Romania, Poland, and Moldova can be classified as high-burden countries, while the remaining countries reported low or sporadic outbreak numbers. This distribution further supports the presence of geographically concentrated endemic areas within the broader study region. Because rabies is a zoonotic disease, human rabies data were included to provide public health context. In Türkiye, WHO Global Health Observatory and WHO Rabies Bulletin Europe records indicated four reported human rabies deaths/cases in 2013 and no reported human cases during 2014–2019; no corresponding WHO GHO data were available for Türkiye for 2020–2023. At the EU/EEA level, ECDC Annual Epidemiological Reports and EFSA/ECDC One Health Zoonoses Reports showed that human rabies/Lyssavirus infections were rare during the study period and were mainly travel-associated. ECDC documented sporadic travel-associated human rabies cases in 2013, 2014, and 2016–2018, while five human Lyssavirus infections were reported in 2019, including one locally acquired European bat lyssavirus 1 (EBLV-1) infection in France. EFSA/ECDC reported no human cases acquired within the EU between 2020 and 2023; in 2023, one human case was reported by France and was probably infected in Morocco.

### 3.2. Descriptive Statistics

Descriptive statistical analysis showed that the mean number of outbreaks per year was 432.27 ± 260.14 (SD), with a median value of 336. The highest number of outbreaks was recorded in 2013 (1022), while the lowest was observed in 2021 (167), indicating considerable interannual variability despite the overall declining trend. The observed difference between the mean and median values, together with the wide range between minimum and maximum values, reflects a non-uniform distribution of outbreaks over time, characterized by periods of both sharp decline and subsequent fluctuation. Descriptive statistics summarizing the annual number of rabies outbreaks are presented in Table 2.

### 3.3. Distribution by Animal Species

To address the animal-species distribution of rabies in Türkiye, species-resolved official surveillance data were examined. In the WHO Rabies Bulletin Europe dataset, records for Türkiye were available for 2013–2019, although the query covered the full study period 2013–2023. Unlike the ADIS dataset, which was used for outbreak-level analysis, the WHO Rabies Bulletin Europe species-resolved data are presented as reported animal rabies cases and were used only for the analytical description of species distribution in Türkiye.

During 2013–2019, 3588 reported animal rabies cases were recorded in Türkiye, excluding human cases. Cattle represented the largest proportion of reported animal cases (1870; 52.1%), followed by dogs (998; 27.8%), foxes (285; 7.9%), goats/sheep (186; 5.2%), cats (147; 4.1%), and equidae (48; 1.3%). Wildlife cases were dominated by foxes, with smaller numbers reported in wolves, martens, badgers, other carnivores, other wildlife, and wild boar. The distribution of reported animal rabies cases by species in Türkiye is presented in Table 3.

The query covered 2013–2023, but species-resolved records for Türkiye were returned for 2013–2019 only. Human cases were excluded from the denominator. The RBE wildlife category excludes bats; bats are reported separately.

Although no bat rabies cases were reported for Türkiye in the RBE records retrieved for 2013–2019, this finding should not be interpreted as evidence that bat-associated lyssaviruses are epidemiologically irrelevant. The RBE wildlife category explicitly excludes bats, and bat-associated lyssaviruses are addressed through separate surveillance frameworks. Additional official and scientific sources were assessed to determine whether bat-associated lyssavirus data could be quantified for Türkiye during the study period. The results of this source-based assessment are presented in Table 4.

The source assessment did not identify a complete, comparable bat-specific quantitative time series for Türkiye for 2013–2023. Therefore, bat-associated lyssaviruses could not be included in the quantitative species-distribution analysis. This finding should be interpreted as a limitation of the available surveillance sources rather than as evidence of absence.

### 3.4. Spatial Distribution and Regional Patterns

To support the analysis of spatial patterns, geographical maps obtained from the Animal Disease Information System (ADIS) were used.

**Figure 1:**
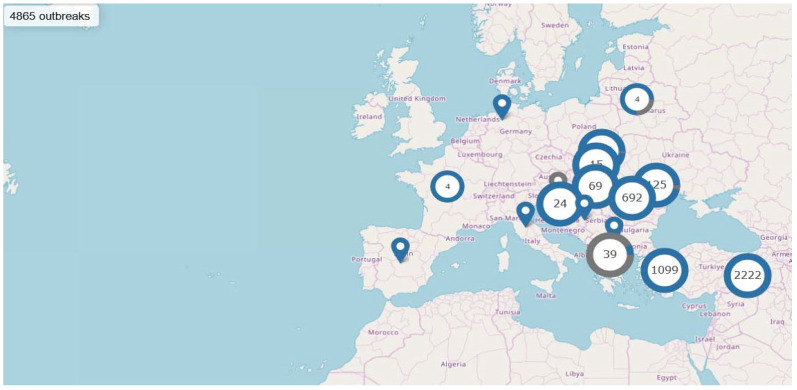
Geographical distribution of rabies outbreaks in Europe and Türkiye (2013–2023), as provided by the Animal Disease Information System (ADIS).

Figure 1 illustrates the geographical distribution of rabies outbreaks across Europe and Türkiye during the period 2013–2023. The spatial pattern demonstrates a clear clustering of outbreaks in Eastern and Southeastern Europe, with the highest concentration observed in Türkiye, Romania, and Moldova. In contrast, Western and Central European countries show predominantly sporadic occurrence, indicating a substantially lower epidemiological burden. This spatial distribution reflects a marked geographical heterogeneity, where a limited number of countries account for the majority of reported outbreaks, while others report only isolated cases. The clustering of outbreaks in specific regions suggests the presence of persistent endemic zones, rather than a uniform distribution of the disease across the study area. The observed spatial pattern indicates that rabies outbreaks are not randomly distributed but are concentrated in regions with sustained transmission over time. These findings support the existence of geographically defined high-risk areas, particularly in Eastern and Southeastern Europe, where the disease remains more consistently reported.

**Figure 2:**
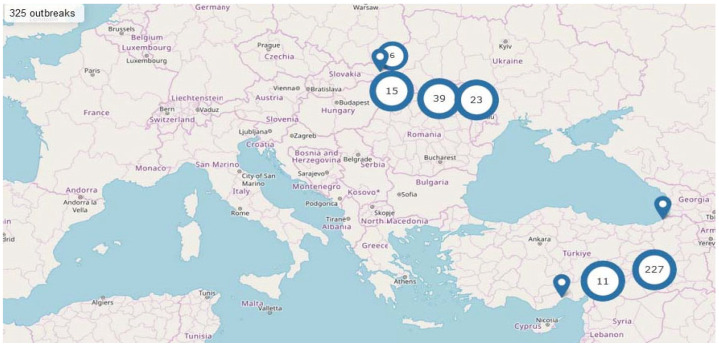
Geographical distribution of rabies outbreaks in Europe in 2023, as provided by the Animal Disease Information System (ADIS).

Figure 2 presents the geographical distribution of rabies outbreaks in Europe in 2023. Only six countries—Moldova, Poland, Romania, Slovakia, Türkiye, and Hungary—reported outbreaks during this year. Compared to the broader spatial distribution observed over the entire study period (Figure 1), this pattern indicates a substantial contraction in the geographical spread of rabies. The restriction of outbreaks to a limited number of countries suggests a reduction in spatial dispersion and a concentration of the disease within specific regions. Despite this contraction, the continued presence of outbreaks in these countries indicates that rabies remains established in defined endemic areas rather than being fully eliminated. This spatial pattern reflects a shift from a more widespread distribution toward a localized persistence of the disease, particularly in Eastern and Southeastern Europe.

**Figure 3:**
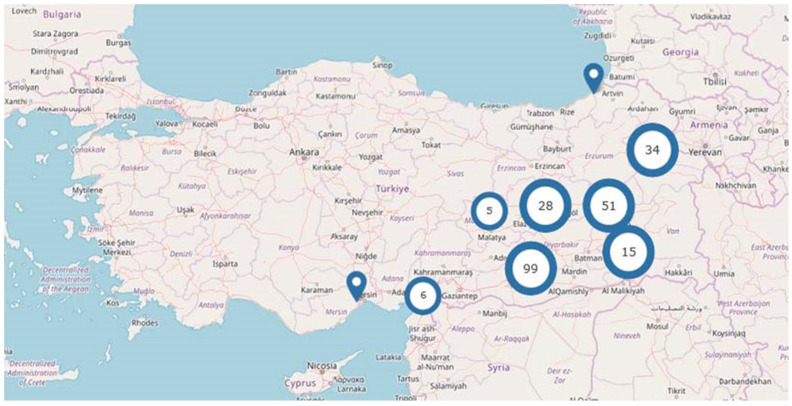
Geographical distribution of rabies outbreaks in Türkiye in 2023, as provided by the Animal Disease Information System (ADIS).

Figure 3 illustrates the regional distribution of rabies outbreaks in Türkiye in 2023. A total of 240 outbreaks were reported, representing 7.2% of all outbreaks recorded in Türkiye during the study period. The spatial pattern shows a clear concentration of outbreaks in the eastern and southeastern regions of the country, while western regions report substantially fewer cases. This distribution indicates pronounced regional heterogeneity in rabies occurrence within Türkiye. The clustering of outbreaks in specific regions suggests the presence of localized areas of sustained transmission rather than a uniform national distribution. This pattern highlights the uneven epidemiological burden within the country and indicates that certain regions may act as persistent hotspots of rabies activity.

**Figure 4:**
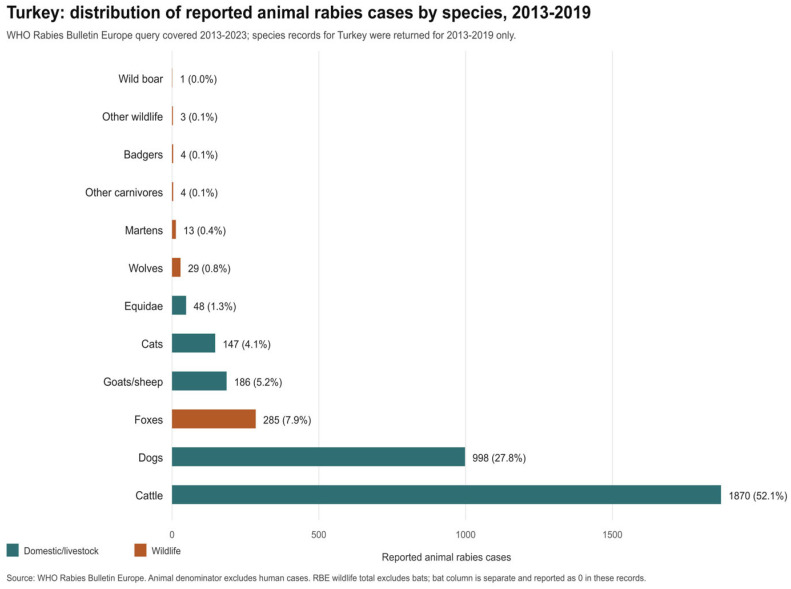
Distribution of reported animal rabies cases by species in Türkiye, 2013–2019. Note: Data are based on WHO Rabies Bulletin Europe species-resolved records. The query covered 2013–2023, but species-level records for Türkiye were returned for 2013–2019 only. Human cases were excluded from the denominator. Percentages are rounded to one decimal place.

To complement the regional map of Türkiye, Figure 4 presents the distribution of reported animal rabies cases by species in Türkiye for 2013–2019. The figure confirms that reported cases were predominantly associated with cattle and dogs, while foxes represented the main wildlife species.

### 3.5. Statistical Analysis of Temporal Trends and Regional Differences

A multiple linear regression model was applied to assess temporal trends and regional differences between Türkiye and the remaining European countries. The model included year, region, and their interaction term. The model was statistically significant (R^2^ = 0.536, *p* = 0.003), indicating that temporal and regional factors explain a substantial proportion of the variability in outbreak numbers. Türkiye had significantly higher annual outbreak counts compared to the European countries (β = 171.55, *p* = 0.007), indicating a markedly higher baseline level of reported outbreaks. A significant decreasing trend over time was observed in the European countries (β = −36.21 outbreaks/year, 95% CI: −69.85 to −2.57, *p* = 0.046, R^2^ = 0.374), while a similar but less pronounced decline was identified in Türkiye (β = −21.30 outbreaks/year, 95% CI: −39.84 to −2.76, *p* = 0.030, R^2^ = 0.425). The interaction between year and region was not statistically significant (*p* = 0.410), indicating that the rate of decline did not differ significantly between Türkiye and the European countries. This suggests that, despite differences in absolute outbreak numbers, the overall temporal trend followed a comparable decreasing pattern in both regions.

A sensitivity analysis excluding Türkiye confirmed that the decreasing trend remained statistically significant (β = −36.21 outbreaks/year, *p* = 0.046), demonstrating that the observed decline is not solely driven by the high number of outbreaks reported in Türkiye. The mean annual number of outbreaks was significantly higher in Türkiye (301.91 ± 108.30) compared to the European countries (130.36 ± 196.36) (*p* = 0.030), further highlighting the substantial regional disparity in rabies occurrence. The results of the regression and comparative analysis of rabies outbreak trends are presented in Table 5.

### 3.6. Annual Percentage Change in Rabies Outbreaks

The annual percentage change in rabies outbreaks during the study period (2013–2023) is presented in Table 6.

The results show considerable year-to-year variability despite the overall decreasing trend. The largest decline occurred between 2015 and 2016 (−62.6%), while the largest increase was observed between 2021 and 2022 (+92.2%). The magnitude of these fluctuations indicates that the temporal dynamics of rabies outbreaks were highly variable and did not follow a consistent linear pattern. Periods of substantial decline were followed by temporary increases, suggesting intermittent changes in outbreak occurrence over time. The average annual change corresponds to a decrease of approximately 11% per year; however, the observed variability indicates that this average value does not fully capture the complexity of the temporal trend.

## 4. Discussion

### 4.1. Temporal Trends and Epidemiological Patterns of Rabies Outbreaks

Rabies continues to represent a significant zoonotic threat to both animal and human health, despite long-standing control efforts in many regions. Although substantial progress has been achieved through vaccination and surveillance programs [32], the disease persists in certain areas where wildlife reservoirs and domestic animal populations facilitate virus circulation [33,34].

Although the present study focuses on animal rabies, the inclusion of human rabies data is essential because the ultimate objective of rabies surveillance and control is the prevention of human exposure and fatal disease.

The findings of the present study demonstrate a clear overall decline in the number of reported rabies outbreaks between 2013 and 2023, with a reduction of approximately 68% over the study period. This decreasing trend was confirmed by the regression analysis, which showed a statistically significant negative association between year and outbreak occurrence. However, the temporal pattern was not uniform, as periods of sharp decline were followed by temporary increases, indicating fluctuating outbreak dynamics rather than a consistent linear decrease. These results provide a more detailed characterization of rabies trends compared to routine surveillance summaries, as the study integrates descriptive epidemiology with statistical modeling and regional comparison. In particular, the identification of non-linear temporal patterns and variability in annual changes highlights the complexity of rabies dynamics across the study region.

### 4.2. Role of Animal Hosts and Transmission Dynamics

From 2013 to 2023, rabies outbreaks mainly affected domestic dogs, wild animals, and cats. Differences among species are associated with behavioral factors, such as aggression in wildlife and stray animals, which facilitate transmission [35]. Domestic dogs in both rural and urban environments, particularly stray populations, often gather in groups where interactions, fights, and competition for food promote virus transmission [36]. Wild animals may increase their foraging activity due to food scarcity, and encounters during hunting or disease-induced aggression can enhance cross-species transmission. Early clinical signs of rabies are often nonspecific and rapidly progress to neurological dysfunction within a few days [6,37]. The variability of the incubation period further complicates disease control [3,38]. The presence of the virus in saliva prior to the onset of clinical signs increases the risk of transmission, particularly following contact with asymptomatic infected animals. Transmission to humans is frequently associated with contact involving infected domestic animals [39]. The findings of the present study confirm that both domestic animals and wildlife contribute to rabies epidemiology, supporting the coexistence of domestic and sylvatic transmission cycles. Domestic dogs were identified as the most frequently affected group, while wildlife species represented a substantial proportion of reported outbreaks. This distribution indicates that, although control measures targeting domestic animals are essential, the persistence of wildlife reservoirs may limit the effectiveness of eradication efforts. The involvement of multiple animal categories suggests that transmission dynamics are complex and cannot be attributed to a single host group. In particular, the presence of cases in both domestic animals and wildlife supports the role of interspecies transmission, which may facilitate the continued circulation of the virus in certain regions despite ongoing control measures. This highlights the need for integrated control strategies addressing both domestic and wildlife populations.

The absence of bat-related data in ADIS should be interpreted cautiously. ADIS is suitable for reported terrestrial animal outbreaks, but it is not an adequate standalone source for assessing bat-associated lyssaviruses. Bats represent a distinct surveillance challenge because lyssavirus detection depends on targeted passive or active bat surveillance, appropriate diagnostic testing, and submission of suspect animals. Consequently, the present study does not conclude that bat-associated lyssaviruses are absent or unimportant in Türkiye. Instead, the lack of bat-specific records is acknowledged as a limitation of the available surveillance systems.

### 4.3. Regional Differences and Challenges in Rabies Control

The results of the present study demonstrate a substantial decline in the number of reported rabies outbreaks in Europe and Türkiye during the period 2013–2023. The total number of outbreaks decreased from 1022 cases in 2013 to 325 cases in 2023, representing an overall reduction of approximately 68%. The established downward trend is consistent with findings from previous epidemiological studies, which attribute the reduction in rabies in Europe to long-term control strategies [40], including systematic vaccination of domestic animals and large-scale oral vaccination (ORV) campaigns targeting wildlife reservoirs, particularly red fox populations [41,42,43]. For several decades, the complex eradication measures led to elimination of sylvatic rabies in many countries in Western and Central Europe [44,45]. The observed decline should not be attributed solely to vaccination programs. The trends identified in the present study may also be influenced by differences in surveillance systems, reporting practices, and diagnostic capacity across countries and over time, which could affect the number of reported outbreaks. This suggests that the reduction reflects not only the success of control measures but also potential variability in detection and reporting. The notable decline in rabies outbreaks persisted until 2021–2022, which saw the highest rates of disease among EU countries. This increase resulted from recurring cases in wild and domestic animals in Poland, Romania, Hungary, and Slovakia—regions that had not reported outbreaks for several years [46]. This re-emergence of new rabies cases in animals coincides with the COVID-19 crisis and post-pandemic period [47], which disturbed to a great extent the essential health services, including the regulated epidemiological measures against contagious animal diseases, as observed also in other geographical areas [48,49]. The COVID-19 pandemic also led to a significant increase in stray dog populations, caused by abandonment and fear of disease transmission to pet owners. This rise likely increased the risk of rabies transmission due to the growing number of free-roaming dogs, which are often aggressive, fight, bite, and lack access to food, healthcare, and supervision [50].

After the pandemic, mandatory dog vaccinations and oral vaccination programs for foxes were resumed as part of regular surveillance and eradication efforts. However, research indicates that effective cooperation between EU and non-EU countries is crucial to building immune barriers within member states and neighboring infected areas, enabling successful, coordinated, and sustainable oral rabies vaccination (ORV) campaigns [46].

These measures have significantly limited the circulation of the virus in several regions and have contributed to the gradual stabilization of the epidemiological situation in Europe. Nevertheless, the persistence of cases in some countries indicates that rabies cannot yet be considered fully eliminated from the region. As found in the present study, the increase in rabies incidence at the end of the investigated period occurred in some member states (Romania, Hungary) sharing a border with third countries such as Ukraine and Moldova, where disruptions of vaccination programmes in wildlife were reported [46,51].

The spatial patterns identified in the present study suggest that rabies outbreaks are not randomly distributed but tend to cluster in geographically connected regions. This supports the potential role of cross-border transmission, particularly in areas where countries with higher outbreak numbers are located in close proximity. Local ecological conditions, wildlife population dynamics, and differences in the management of stray animal populations may influence the continued occurrence of sporadic outbreaks [52,53]. Moreover, among the risk factors for re-emergence of rabies in European territories are movements of people and animals to and from endemic regions, illegal animal transportation, and inconsistencies in vaccination programmes [54,55].

A key finding of the present study is the pronounced regional disparity between Türkiye and European Union countries. Türkiye accounted for the majority of reported outbreaks, indicating a substantially higher epidemiological burden compared to the EU. This observation is supported by the statistical analysis presented in the Results section, which demonstrated significantly higher annual outbreak numbers in Türkiye, while both regions exhibited a similar decreasing trend over time. This indicates that, despite differences in absolute numbers, the underlying temporal dynamics are comparable, and the overall decline is not solely driven by a single country.

Vaccination remains the most effective measure for the prevention and control of rabies [56]. High-quality vaccine baits have proven effective in controlling and managing fox-borne rabies in western and central Türkiye. In Türkiye, the zoonosis is maintained by dogs and red foxes, and rabies cases in animals decreased from 2013 to 2022. Starting with a peak of 1022 outbreaks reported in 2013, the number fell to 167 in 2021 and then it rose again to 321 in 2022, with similar trends observed in other epidemiological studies [57]. Initially, cases were mainly reported in the western part of the country, with the east and south following later. The decline is likely due to the effects of an active oral vaccination program for wildlife rabies control in the west.

Rabies in wildlife in Türkiye is rarely reported, with red foxes (Vulpes vulpes) and golden jackals (Canis aureus) being the most common species documented. Phylogenetic analyses showed that viruses from these species were closely related, suggesting cross-species transmission facilitated by foxes as the cause of canine rabies in the region [58,59,60]. Spatial tracking shows cattle rabies influenced by foxes, as past cases also indicate fox-mediated rabies [61].

Taking into consideration the inter-host genetic diversity in rabies virus [62], especially the transmission from wildlife to domestic animals, large-scale ORV campaigns were carried out between 2019 and 2022, covering about 25–30% of Turkish areas with key epidemiological importance [57]. Following these efforts, rabies cases in foxes dropped significantly. In 2023, sporadic cases persisted in vaccinated regions, mainly in southeastern Türkiye involving cattle, dogs, donkeys, foxes, goats, sheep, and wolves, as reported in the present study. While the regulatory framework recommends the first round of the two-phase distribution of the ORV baits to be carried out in spring when young animal populations rise [25], it might be more effective to spread them earlier, in February, during the pre-spring period, when temperatures remain stable, as seen in Türkiye [57]. Under the current climate change, in Bulgaria, the first phase of the oral vaccination in foxes is carried out in March–April, ensuring sustained immune belts near the borders with Romania and Türkiye as neighbouring countries with far more outbreaks than Bulgaria itself during the last years [63].

The findings of the present study also emphasize the role of domestic animals as a potential bridge between wildlife reservoirs and human exposure. Although wildlife species are often involved in maintaining the virus in the environment, contacts between infected wild animals and domestic dogs or livestock can facilitate further spread of the disease [61]. For this reason, regular vaccination of companion animals [6] and farm animals [64] remains an essential component of rabies prevention strategies.

In the fight against rabies, efforts are also focused on increasing knowledge and awareness among animal owners, healthcare workers and the public as a whole. In several studies, rabies knowledge and practices were assessed among dog owners [65], public interactions with stray dog populations and health concerns [66,67] and even among medical students [68]. Within these studies it became clear that for the purpose of rabies control and eradication it is crucial to implement strategies such as addressing knowledge gaps through vaccination and organizing educational campaigns for the relevant stakeholders.

In rabies-endemic regions, ZeroRabiesApp (ZRA), a web-based platform, has been incorporated with post-bite prophylaxis guidelines to enhance healthcare effectiveness and raise awareness among health professionals about rabies management [69]. The app provides access to the latest guidelines from the WHO and CDC, supports rabies treatment decision-making, and offers a database of biological drugs, vaccines, and other products for rabies prevention. It can generate personalized prophylaxis plans for users. Such technological innovations are especially valuable in regions where biological products are limited and rabies remains endemic, and they may also support epidemiological modelling and preparedness strategies [70].

## 5. Limitations

This study has several limitations that should be considered when interpreting the results. The analysis is based on secondary surveillance data obtained from the Animal Disease Information System (ADIS) and, where applicable, the World Animal Health Information System (WAHIS). Differences in reporting practices, surveillance intensity, and diagnostic capacity between countries may have influenced the number of reported outbreaks. To ensure consistency and avoid duplication, a single primary data source (ADIS) was used for quantitative analysis, and no merging of records between ADIS and WAHIS was performed. However, potential inconsistencies between the two systems cannot be fully excluded. The use of reported outbreak data instead of incidence rates relative to animal population size limits direct comparisons between countries. The absence of molecular or phylogenetic data in the present study also restricts the ability to assess transmission pathways and virus circulation patterns in detail. A limitation of the analysis is the heterogeneity of official surveillance sources. ADIS provided outbreak-level information but did not provide a complete species-resolved dataset suitable for assessing bat-associated lyssaviruses. The WHO Rabies Bulletin Europe provided species-resolved data for Türkiye only for 2013–2019, despite querying the 2013–2023 period. The species-distribution analysis was restricted to the years for which official species-level data were available. The absence of bat records in the analysed animal dataset should not be interpreted as evidence of absence of bat-associated lyssavirus circulation or public health relevance. Consequently, conclusions on species distribution mainly reflect reported terrestrial animal rabies and should not be extended to bat lyssavirus ecology. The reliance on officially reported outbreaks may also result in underestimation of the true burden of the disease, particularly in regions with limited surveillance capacity. Data from the European Centre for Disease Prevention and Control (ECDC) were used to contextualize the public health relevance of rabies in humans. However, due to fundamental differences in surveillance objectives, case definitions, and populations under observation, ECDC data are not directly comparable with animal health data obtained from ADIS and WAHIS, but rather complement the epidemiological interpretation.

## 6. Conclusions

Rabies remains a deadly zoonotic disease; however, the results of the present study demonstrate a significant decrease in reported outbreaks across Europe and Türkiye over the past decade. This decline, quantified as approximately 68%, reflects the impact of long-term vaccination programs, improved surveillance, and coordinated control strategies. Despite this progress, rabies has not been fully eradicated and continues to pose a risk to both animal and public health. The study identified pronounced regional disparities, with Türkiye accounting for a substantially higher number of reported outbreaks compared to European Union countries, although both regions exhibited similar decreasing temporal trends. The findings also highlight that rabies dynamics are not uniform over time, with periods of decline interrupted by temporary increases, indicating the influence of multiple epidemiological factors. In addition to vaccination, these trends may be affected by variations in surveillance systems, reporting practices, and cross-border transmission. The coexistence of domestic and wildlife transmission cycles, as demonstrated in this study, underscores the need for integrated control strategies targeting both animal populations. Sustained vaccination of domestic animals, continued oral vaccination of wildlife, and strengthened cross-border cooperation remain essential for long-term control. The results emphasize that effective rabies control requires a coordinated One Health approach, combining veterinary, public health, and wildlife management efforts to reduce the risk of disease persistence and re-emergence.

## Figures and Tables

**Table 1:** Reported number of animals linked to rabies outbreaks across European countries and Türkiye from 2013 to 2023.

Country	2013	2014	2015	2016	2017	2018	2019	2020	2021	2022	2023	Total for the Period	Percentage of Total Outbreaks
Bosnia and Herzegovina								1				1	0.02%
Bulgaria		1										1	0.02%
Croatia	23	1										24	0.49%
France	1		2					1		1		5	0.10%
Germany									1			1	
Greece	28	11										39	0.80%
Hungary	24	23		1	2					4	15	69	1.42%
Italy								1				1	0.02%
Lithuania	1		2			1						4	0.08%
Moldova								70	19	13	23	125	2.57%
Poland	192	92	95	15	3	4	1	7	112	37	7	565	11.61%
Romania	395	133	67	15	2	3	4	5	3	26	39	692	14.22%
Slovakia	7		5							2	1	15	0.31%
Slovenia		1										1	0.02%
Spain	1											1	0.02%
Türkiye	350	530	401	183	331	308	347	251	142	238	240	3321	68.26%
Total outbreaks by year	**1022**	**792**	**572**	**214**	**338**	**316**	**352**	**336**	**167**	**321**	**325**	**4865**	

Source: Animal Disease Information System (ADIS), European Commission. Note: The values presented in the row “Total outbreaks by year” correspond to the annual totals.

**Table 2:** Descriptive statistics of annual rabies notifications (2013–2023).

Study Period	Total Outbreaks	Mean Outbreaks per Year	Median	Standard Deviation	Minimum	Maximum
2013–2023	4865	432.27	336	260.14	167	1022

**Table 3:** Distribution of reported animal rabies cases by species in Türkiye, 2013–2019.

Species	Category	Cases	% of Animal Cases
Cattle	Domestic/livestock	1870	52.1
Dogs	Domestic	998	27.8
Foxes	Wildlife	285	7.9
Goats/sheep	Livestock	186	5.2
Cats	Domestic	147	4.1
Equidae	Livestock	48	1.3
Wolves	Wildlife	29	0.8
Martens	Wildlife	13	0.4
Badgers	Wildlife	4	0.1
Other carnivores	Wildlife	4	0.1
Other wildlife	Wildlife	3	0.1
Wild boar	Wildlife	1	<0.1
Bats	Bats	0	0.0
**Total animal cases**		**3588**	**100.0**

Source: WHO Rabies Bulletin Europe (Available online: https://www.who-rabies-bulletin.org/, accessed on 15 January 2026).

**Table 4:** Source-based assessment of bat-associated lyssavirus data availability for Türkiye, 2013–2023.

Source	Coverage Checked	Finding	Use in Manuscript
ADIS	Bat-specific outbreak information	No bat-specific records were available in the analysed ADIS extract.	Not used to infer absence of bat-associated lyssaviruses.
WHO Rabies Bulletin Europe	Species-resolved rabies records, including bat column	Türkiye records were available for 2013–2019; bat column = 0; no Türkiye species records were returned for 2020–2023.	Used for animal species distribution, with explicit limitations.
ECDC Annual Epidemiological Reports	Human rabies/Lyssavirus and EBLV context	Documents rare human Lyssavirus infections and the relevance of European bat lyssaviruses in Europe.	Used for public health and bat-lyssavirus context.
EFSA/ECDC One Health Zoonoses Reports	EU-level animal and human rabies/Lyssavirus surveillance	Reports bat surveillance and rare spillover events in Europe.	Used to show that bat-associated lyssaviruses remain epidemiologically relevant.
WOAH/WAHIS public outputs	Public rabies event and summary reports	No complete comparable bat-specific Türkiye series for 2013–2023 was identified.	Consulted but not used quantitatively.
Published literature on Türkiye	Türkiye-specific rabies epidemiology and bat discussion	Reports historical bat rabies evidence and limited bat surveillance in Türkiye.	Used to support the limitation and the need for targeted bat surveillance.

**Table 5:** Regression and comparative analysis of rabies outbreak trends (2013–2023).

Analysis	β (Outbreaks/Year)	95% CI	R^2^	*p*-Value
EU (excluding Türkiye)	−36.21	−69.85 to −2.57	0.374	0.046
Türkiye	−21.30	−39.84 to −2.76	0.425	0.030
Combined model	—	—	0.536	0.003
Region effect (Türkiye vs. EU)	+171.55	—	—	0.007
Interaction (year × region)	—	—	—	0.410

**Table 6:** Annual percentage change in rabies outbreaks (2013–2023).

Year	Number of Outbreaks	Annual Percentage Change (%)
2013	1022	–
2014	792	−22.5
2015	572	−27.8
2016	214	−62.6
2017	338	+57.9
2018	316	−6.5
2019	352	+11.4
2020	336	−4.5
2021	167	−50.3
2022	321	+92.2
2023	325	+1.2

## Data Availability

The original contributions presented in this study are included in the article. Further inquiries can be directed to the corresponding author.
